# Epidemiology and clinical outcomes of clinically suspected multiple endocrine neoplasia type 1 in South Korea: a nationwide cohort study

**DOI:** 10.3389/fendo.2025.1562282

**Published:** 2025-06-18

**Authors:** Kyoung Jin Kim, Min Heui Yu, Yoon-a Hwang, Shinje Moon, Namki Hong, Yumie Rhee

**Affiliations:** ^1^ Division of Endocrinology and Metabolism, Department of Internal Medicine, Korea University College of Medicine, Seoul, Republic of Korea; ^2^ Department of Internal Medicine, Yonsei University College of Medicine, Seoul, Republic of Korea; ^3^ Severance ENdocrinology daTa scIeNcE pLatform (SENTINEL) Team, Division of Endocrinology, Department of Internal Medicine, Yonsei University College of Medicine, Seoul, Republic of Korea; ^4^ Department of Internal Medicine, Hanyang University College of Medicine, Seoul, Republic of Korea

**Keywords:** multiple endocrine neoplasia type 1, epidemiology, comorbidity, cohort study, mortality

## Abstract

**Background:**

Multiple endocrine neoplasia type 1 (MEN1) is a rare hereditary disorder characterized by multiorgan endocrine tumors, primarily affecting the parathyroid glands, pituitary, and pancreas. Despite its clinical significance, the epidemiology and outcomes of clinically suspected MEN1 in Asian populations remain limited. This study aimed to investigate the prevalence, comorbidities, and mortality risk associated with clinically suspected MEN1 in South Korea.

**Methods:**

We conducted a retrospective cohort study using the Korean National Health Insurance Service database (2003–2020), identifying clinically suspected MEN1 cases via two operational definitions: (1) ICD-10 MEN1 code (D44.8) with medical service records and (2) diagnoses or interventions for at least two MEN1-associated conditions (primary hyperparathyroidism, pituitary adenoma, or duodenopancreatic neuroendocrine tumors). Cases (n = 412) were matched 1:10 with controls (n = 4,120) by age, sex, and index year. Clinical characteristics, comorbidities, and mortality were analyzed using Kaplan–Meier survival analysis and multivariable Cox regression.

**Results:**

The incidence of clinically suspected MEN1 peaked in individuals aged 40–49 years, with a higher prevalence in females (64.6%). Parathyroid involvement was the most common manifestation (58.6%), followed by pituitary (22.3%) and duodenopancreatic tumors (19.9%). Comorbidities, including diabetes mellitus (22.6%), hypertension (38.1%), and dyslipidemia (20.6%), were significantly more prevalent in MEN1 patients than controls. Mortality was elevated among MEN1 patients (HR 3.69; 95% confidence intervals (CI) 2.56–5.31), particularly those with multiorgan involvement, although hazard ratios varied by organ combination and had wide, overlapping CIs. The mean age at death was significantly younger in MEN1 patients (60.1 years) than in controls (68.0 years).

**Conclusions:**

This nationwide cohort study of clinically suspected MEN1 in South Korea reveals a substantial clinical burden, particularly among patients with multiorgan involvement. Enhanced clinical surveillance and early interventions are essential to improve outcomes for MEN1 patients. Future research integrating genetic testing and clinical data is needed to further guide management strategies.

## 1 Introduction

Multiple endocrine neoplasia type 1 (MEN1) is a rare yet clinically significant endocrine disorder with a global prevalence of approximately 1–20 per 100,000 individuals (1, 2). MEN1 is characterized by tumors in multiple endocrine glands, affecting the parathyroid glands, anterior pituitary, and pancreatic islet cells (2). This is caused by mutations in *MEN1*, which encodes menin, a crucial regulator of cell growth and proliferation (3, 4). The epidemiology of MEN1 varies regionally in terms of clinical manifestations and genetic mutations, which are crucial for understanding the impact of the disease and for guiding management strategies (5). Given the complexity of MEN1 and the risk of significant morbidity from undiagnosed tumors, diagnostic tools and structured screening guidelines are essential for timely tumor detection and effective clinical management (6).

The epidemiology of MEN1 remains under-researched, as it has largely been restricted to a few national cohorts (7–10). These studies have offered essential preliminary insights into the demographic and clinical variabilities of the condition, such as differential age of onset, sex predisposition, and its endocrinologic presentation (1). Some cohorts reported a female predominance while others presented a more balanced distribution between the sexes (8, 11). These reports highlight the clinical complexity of MEN1, which is expressed in a range of conditions, from primary hyperparathyroidism (PHPT) to neuroendocrine tumors, each with its own prognostic and therapeutic challenges. Despite significant advancements in detection technologies such as genetic testing, MEN1 remains underdiagnosed (1, 12). This issue is particularly evident in Asia, where the lack of substantial epidemiological data further limits a thorough assessment of its clinical impact (13).

Current research on MEN1 in South Korea, has been limited to isolated case reports and cross-sectional studies from a few institutions (14). To expand this limited understanding, our study utilized comprehensive data from the Korean National Health Insurance Service (NHIS) to conduct the first nationwide cohort study of clinically suspected MEN1. This initiative was designed to evaluate the prevalence, demographic patterns, and clinical features of clinically suspected MEN1, thereby enhancing our understanding of MEN1.

## 2 Materials and methods

### 2.1 Data source

This study was based on the Korean NHIS database, which includes health information for 51.5 million residents, representing approximately 97% of South Koreans (15). This database serves as a resource for health reimbursement and includes records from 2002 to 2020. The NHIS database contains demographic details, hospital admissions, diagnostic codes according to the International Classification of Diseases (10th revision; ICD-10), prescriptions, medical procedures, and mortality data reported by healthcare providers. Our analysis covers the period from 2003 to 2020.

### 2.2 Study population

Precise identification of clinically suspected MEN1 cases is critical because of the non-specificity of the ICD-10 code D44.8, which encompasses all MEN syndromes. In this study, we established two operational definitions to ensure accurate selection of clinically suspected MEN1 cases. The first definition required the D44.8 code (multiple endocrine adenomatosis) in at least one medical service record, recognizing this as the principal diagnosis. Stringent criteria were applied to exclude conditions potentially confounding the diagnosis of clinically suspected MEN1, such as medullary thyroid carcinoma (MTC) and end-stage renal disease requiring dialysis before the index year, thereby enhancing the specificity of our cohort. For MTC, without a specific ICD-10 code, it was defined as thyroid cancer (C73) diagnosed twice, surgery within 2 years, with post-surgical measurements of calcitonin twice and carcinoembryonic antigen once, measured concurrently with calcitonin. The second definition included patients with two or more medical interventions or diagnoses associated with clinically suspected MEN1-related tumor. This approach aimed to include a cohort reflecting the full clinical spectrum and diagnostic criteria of clinically suspected MEN1, resulting in a total of 412 patients (**Supplementary Figure S1**).

These operational definitions were validated within our tertiary-level institution, Yonsei University Severance Hospital, providing a sensitivity of 98.9% and a positive predictive value of 73.3%. The focus on high sensitivity was necessary due to the rare nature of clinically suspected MEN1 and the need to capture true cases. For comparison, a control group was matched at 1:10 by age, sex, and index year, with those who did not meet any criteria for clinically suspected MEN1. The index date was defined as the initial recording of relevant diagnostic codes for individuals satisfying the inclusion criteria.

### 2.3 Study outcomes and covariates

To identify the clinical manifestations of clinically suspected MEN1, including PHPT, pituitary adenoma, and duodenopancreatic neuroendocrine tumors, we used ICD-10 diagnostic codes with at least one medical claim and at least one procedural or pharmaceutical code as detailed in **Supplementary Table S1**. Comorbidities, including diabetes mellitus, hypertension, dyslipidemia, osteoporosis, cardiocerebrovascular disease, osteoporotic fractures, and cancer, were identified using corresponding ICD-10 codes recorded at least twice, alongside related medication prescriptions around the index date, as detailed in **Supplementary Table S1**. Additionally, associated conditions such as adrenal involvement, thymic abnormalities, lipomas, central nervous system tumors, and solid tumors were identified using at least two diagnostic codes, and concurrent prescription medication use throughout the study period was also evaluated (**Supplementary Table S1**). Socioeconomic status was classified according to the total national health insurance premiums paid, dividing patients into three groups: lowest 30%, middle 40%, and highest 30%.

### 2.4 Statistical analysis

Data are presented as mean ± standard deviation (SD) for continuous variables, and as counts and percentages for categorical variables. Age-standardized incidence rates of clinically suspected MEN1 were calculated by dividing the number of new cases in a specific age group by the corresponding age-specific national population, with the rates presented per 100,000 persons. We employed Kaplan–Meier analysis to estimate the cumulative incidence of mortality among patients with clinically suspected MEN1 compared with their controls. The log-rank test was used to assess the significance of differences in survival data, and hazard ratios (HRs) with 95% confidence intervals (CIs) were computed to quantify the relative hazard for mortality. In our primary analysis, differential clinical presentations in patients with clinically suspected MEN1 were compared with their controls, considering various confounders, including age, sex, and the Charlson comorbidity index (CCI). Further analyses compared the mortality risks based on the number of affected organs in patients with clinically suspected MEN1. Clinical outcomes in patients with clinically suspected MEN1 were evaluated against those in the control group after excluding any prior history of each outcome. All analyses were further adjusted for confounders including age, sex, and CCI.

Significance was set at a P-value of <0.05. All statistical analyses were conducted using SAS software (version 9.4; SAS Institute Inc., Cary, NC, USA).

## 3 Results

### 3.1 Epidemiology of clinically suspected MEN type 1 by operational definition

In this nationwide cohort study, we analyzed the age at diagnosis and the sex distribution of 412 patients with clinically suspected MEN1 in South Korea. The age at diagnosis varied significantly, with the highest number of cases identified in the 40–49 age group (95 cases), followed by that in the 30–39 (86 cases) and 50–59 (64 cases) age groups. The incidence of clinically suspected MEN1 was notably lower in younger age groups, indicating that MEN1 is more commonly diagnosed in middle-aged adults. The demographic analysis revealed a higher prevalence in females (64.6%) than in males (35.4%) (**Supplementary Figure S2**).

**Figure 1:**
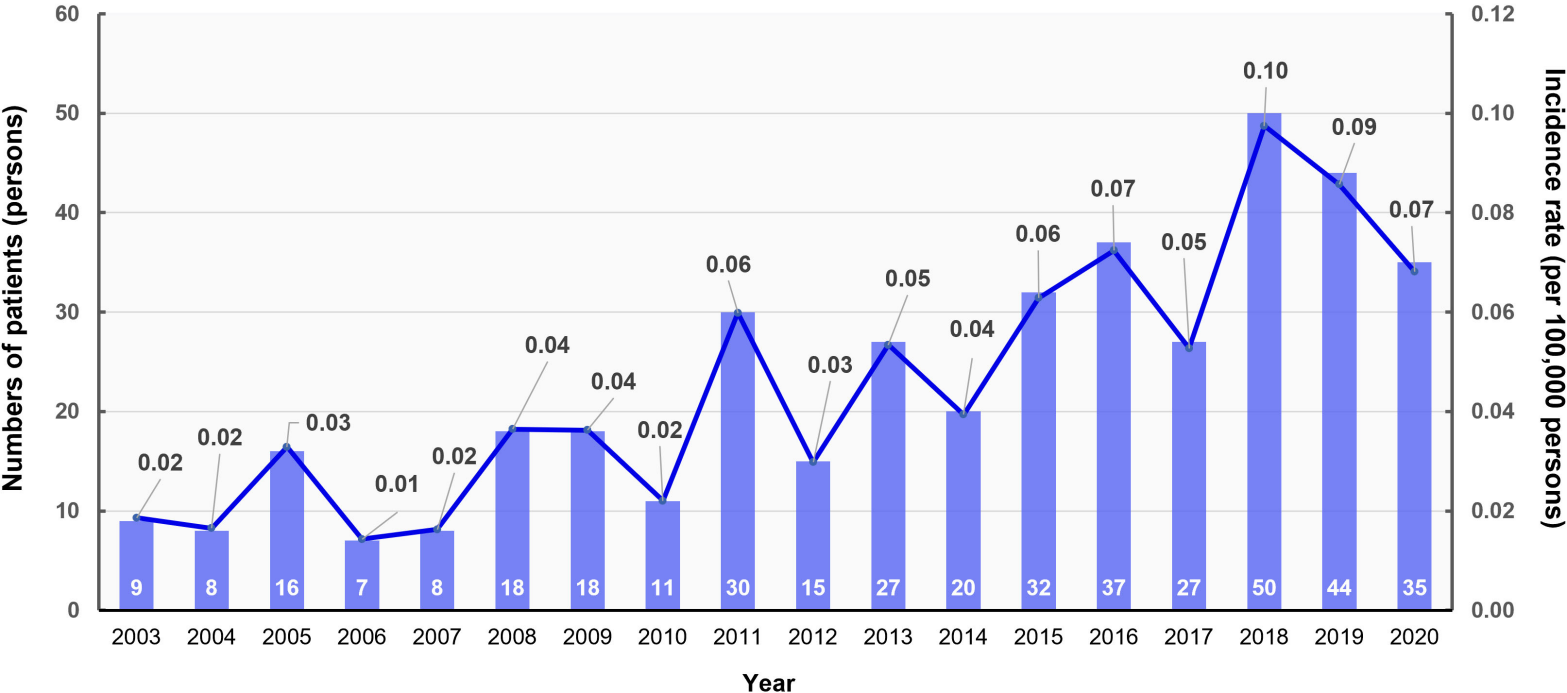
Annual age-standardized event rate and number of patients with multiple endocrine neoplasia type 1 (MEN1) by index year, 2003–2020. The bar graph shows the number of patients diagnosed with MEN1 each year (left y-axis), while the line graph indicates the age-standardized incidence rate per 100,000 persons (right y-axis).

The annual age-standardized event rate and number of patients diagnosed with clinically suspected MEN1 per index year from 2003 to 2020 are depicted in **Figure 1**. The incidence rate per 100,000 persons varied over the years, with a noticeable peak in 2011 (0.06 per 100,000 persons), a significant increase in 2018 (0.10 per 100,000 persons), and a slight decrease in subsequent years. We observed an overall upward trend in the incidence rates, suggesting that improvements in genetic testing may enhance our ability to detect clinically suspected MEN1. Despite this increasing trend, the overall prevalence remains low at 0.87 cases per 100,000, which may indicate underdiagnosis.

### 3.2 Clinical characteristics of with clinically suspected MEN type 1

**Table 1** summarizes the clinical characteristics of 412 patients with clinically suspected MEN1. The mean age at baseline was 43.6 years, and males were slightly younger than females. Parathyroid involvement was seen in 58.5% of patients, often with multiple gland involvement requiring extensive surgeries. Pituitary involvement was noted in 22.3% of patients, with hyperprolactinemia being the most frequent. Duodenopancreatic involvement was observed in 19.9% of patients, with gastrinomas and insulinomas being the most common tumors identified. Among patients with clinically suspected MEN1 who exhibited duodenopancreas involvement, 76 out of 82 patients (92.6%) underwent any pancreatectomy.

**Table 1:** Clinical characteristics and treatment approaches of patients with type 1 MEN.

Variables by organ involvement and treatment	MEN type 1		
	Total (412)	Men (146)	Women (266)
Age at baseline, years, mean (SD)	43.6 ± 15.8	42.5 ± 15.9	44.2 ± 15.7
Parathyroid involvement, n (%)	241 (58.5)	91 (62.3)	150 (56.3)
Treatment			
Single	109	43	66
More than single	155	54	101
More than twice (different time)	38	16	22
Pituitary involvement, n (%)	92 (22.3)	37 (25.3)	55 (20.7)
Hyperprolactinemia	36	9	27
Acromegaly	16	9	7
Cushing’s disease	5	1	4
Treatment			
Surgery for pituitary tumor removal	52	19	33
Caverlactin, bromocriptine, somatostatin analogue	57	27	30
Duodenopancreas involvement, n (%)	82 (19.9)	39 (26.7)	43 (16.2)
Gastrinoma	12	6	6
Insulinoma	6	3	3
Others (VIPoma, or somatostatin secreting)	5	5	0
liver metastasis	12	8	4
Treatment			
Any pancreatectomy	76	34	42
Total pancreatectomy	5	3	2
Partial pancreatectomy	55	23	32
Hepatectomy	4	1	3

MEN, multiple endocrine neoplasia; N, number; SD, standard deviation.

The number of patients listed under parathyroid involvement, pituitary involvement, and duodenopancreas involvement represents unique patient counts. In contrast, numbers indicated under specific treatment categories (e.g., single or multiple parathyroidectomy, pituitary surgery, pancreatic surgery) represent the total number of procedures or interventions performed, allowing multiple counts per patient. Consequently, the sum of procedures or interventions may exceed the number of unique patients with involvement.

### 3.3 Comorbidity and mortality risks in clinically suspected MEN type 1

**Table 2** presents the clinical presentations in patients with clinically suspected MEN type 1 compared to their controls. The Charlson comorbidity index was significantly higher in patients with clinically suspected MEN1 than in controls (2.5 ± 2.5 vs. 0.5 ± 1.1, p < 0.001), indicating that clinically suspected MEN1 patients had a fivefold higher comorbidity burden. Patients with clinically suspected MEN1 had significantly higher rates of comorbidities, including diabetes, hypertension, dyslipidemia, cardiovascular disease, and osteoporotic fractures, compared to controls. Adrenal involvement, particularly adrenal adenomas, was common among these patients. The incidence of cancer was markedly higher in the clinically suspected MEN1 group, with increased rates of thyroid, pancreatic, and liver cancers.).

**Table 2:** Differential clinical presentations in MEN type 1 compared with age- and sex-matched controls.

Variables	MEN type 1	Control	p-value
	(n = 412)	(n = 4,120)	
Age at baseline, years, mean (SD)	43.6 ± 15.8	43.7 ± 15.5	0.868
Age group, n (%)			0.644
0–9	9 (2.2)	40 (1.0)	
10–19	20 (4.9)	250 (6.1)	
20–29	52 (12.6)	520 (12.6)	
30–39	84 (20.4)	840 (20.4)	
40–49	86 (20.9)	860 (20.9)	
50–59	95 (23.1)	950 (23.1)	
60–69	47 (11.4)	470 (11.4)	
70–85	18 (4.4)	180 (4.4)	
>85	1 (0.2)	10 (0.2)	
Socioeconomic status			0.623
Lowest	124 (30.3)	1141 (28.1)	
Middle	112 (27.4)	1158 (28.5)	
Highest	173 (42.3)	1768 (43.5)	
Charlson comorbidity index	2.5 ± 2.5	0.5 ± 1.1	<0.001
Comorbidities			
Diabetes mellitus	93 (22.6)	198 (4.8)	<0.001
Hypertension	157 (38.1)	677 (16.4)	<0.001
Dyslipidemia	85 (20.6)	463 (11.2)	<0.001
Osteoporosis	34 (8.3)	37 (0.9)	<0.001
Cardiovascular disease	47 (11.4)	151 (3.7)	<0.001
Osteoporotic fracture	3 (0.7)	66 (1.6)	0.167
Cancer	216 (52.4)	65 (1.6)	<0.001
Associated diseases			
Adrenal involvements	143 (34.7)	5 (0.1)	<0.001
Adrenal adenoma	130 (31.6)	4 (0.1)	<0.001
Adrenocortical carcinoma	7 (1.7)	0 (0.0)	<0.001
ACTH-independent Cushing syndrome	21 (5.1)	1 (0.02)	<0.001
Primary aldosteronism	3 (0.73)	0 (0.0)	<0.001
Pheochromocytoma	32 (7.8)	0 (0.0)	<0.001
Thymus benign tumors	8 (1.9)	0 (0.0)	<0.001
Lipoma	60 (14.6)	0 (0.0)	<0.001
CNS tumors			
Meningioma	7 (1.7)	0 (0.0)	<0.001
Neoplasm of uncertain or unknown behavior of brain and central nervous system including ependymoma	6 (1.5)	6 (0.2)	<0.001
Solid tumors			
Thyroid cancer (C73)	168 (40.8)	3 (0.1)	<0.001
Pancreatic cancer (C25)	133 (32.3)	16 (0.4)	<0.001
Colorectal cancer (C18–C20)	46 (11.2)	45 (1.1)	<0.001
Liver cancer (C22)	24 (5.8)	75 (1.8)	<0.001
Lung cancer (C34)	22 (5.3)	39 (1.0)	<0.001
Ovarian cancer (C56)	17 (4.1)	33 (0.8)	<0.001
Thymic cancer (C37)	19 (4.6)	0 (0.0)	<0.001
Gastric cancer (C16)	16 (3.9)	37 (0.9)	<0.001
Prostate cancer (C61)	11 (2.7)	34 (0.8)	<0.001
Breast cancer (C50)	9 (2.2)	50 (1.2)	0.097
Uterus cancer (C54, C55)	8 (1.9)	17 (0.4)	<0.001
Kidney cancer (C64)	6 (1.5)	10 (0.2)	<0.001
Cervical cancer (C53)	3 (0.7)	19 (0.5)	0.497
Esophageal cancer (C15)	2 (0.5)	1 (0.02)	0.001
Bladder cancer (C67)	3 (0.7)	10 (0.2)	0.079

MEN, multiple endocrine neoplasia; N, number; SD, standard deviation.

**Figure 2:**
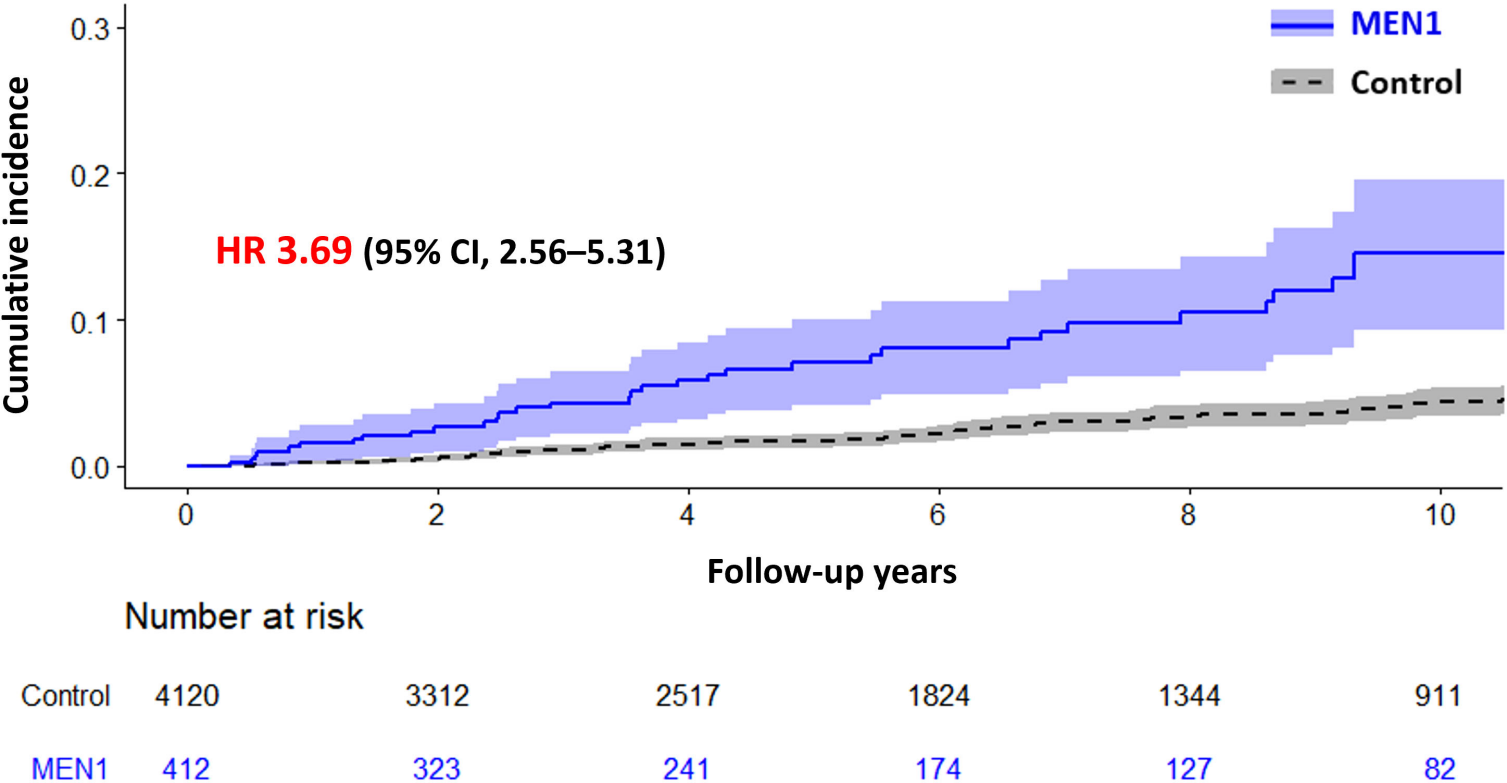
Cumulative Incidence of Mortality in Patients with Multiple Endocrine Neoplasia Type 1 (MEN1) Compared with a Control Group. The graph shows the cumulative incidence of mortality over time for patients with MEN1 versus a control group, with hazard ratio (HR) of 3.69 (95% CI, 2.56–5.31), indicating a significantly higher mortality risk in the MEN1 group. Shaded areas represent the 95% confidence intervals.

**Figure 2** illustrates the cumulative incidence of mortality in patients with clinically suspected MEN1 compared with controls, showing a significantly higher mortality in the clinically suspected MEN1 group (HR, 3.69; 95% CI, 2.56–5.31). The median observation periods were 4.84 (interquartile range [IQR] 2.12–9.21) years for the clinically suspected MEN1 group and 5.29 (IQR, 2.36–9.54) years for the control group, with an overall median of 5.24 (IQR, 2.32–9.54) years. Additionally, the mean age of death for patients with clinically suspected MEN1 was 60.1 ± 14.6 years, which was significantly younger than the control group, which had a mean age of death of 68.0 ± 13.4 years.

As shown in **Table 3**, patients with parathyroid involvement alone had an adjusted HR of 2.73 (95%CI, 1.63–4.56) for mortality. Patients with tumors involving two MEN1-related organ sites demonstrated higher adjusted HRs, though the magnitude varied according to specific organ combinations: 2.82 (95%CI, 1.01–7.83) for parathyroid plus duodenopancreatic involvement; 3.63 (95% CI 1.83–7.20) for parathyroid plus pituitary involvement; and 7.29 (95%CI, 2.54–20.88) for pituitary plus duodenopancreatic involvement. However, the small number of events and wide, overlapping CIs limit clear distinctions among these combinations.

**Table 3:** Comparative mortality risks based on the number of affected organs in patients with MEN type 1.

Patient groups based on organ involvement	Mortality event N (IR)	Univariable HR (95%CI)	Multivariable* HR (95%CI)
Control	76 (4.24)	1 (Ref)	1 (Ref)
Single involvement in			
Parathyroid	23 (14.52)	3.42 (2.13–5.47)	2.73 (1.63–4.56)
Pituitary	17 (22.43)	4.77 (2.68–8.47)	4.67 (2.38–9.13)
Duodenopancreas	12 (19.61)	5.38 (2.69–10.76)	4.04 (1.77–9.19)
Any more than two			
Parathyroid & pituitary	10 (23.76)	5.31 (2.74–10.29)	3.63 (1.83–7.20)
Parathyroid & duodenopancreas	4 (14.45)	3.37 (1.23–9.21)	2.82 (1.01–7.83)
Pituitary & duodenopancreas	4 (47.17)	10.36 (3.7–28.34)	7.29 (2.54–20.88)
All of three			
Parathyroid & pituitary & duodenopancreas	2 (8.54)	1.93 (0.47–7.85)	2.27 (0.53–9.69)

*Adjusted for age, sex, and CCI.

CI, confidence interval; HR, hazard ratio; IR, incidence rate; MEN, multiple endocrine neoplasia; N, number.

In the detailed analysis excluding subjects with a history of the relevant outcomes, patients with clinically suspected MEN1 had significantly higher cumulative incidences of composite cardiovascular events (HR, 2.26), cancer (HR, 9.46), diabetes mellitus (HR, 7.79), anxiety (HR, 2.44), and depression (HR, 2.79) compared with controls, with no significant difference observed for fractures (HR, 2.50) (**Supplementary Figure S3**).

## 4 Discussion

In this nationwide cohort study of 412 individuals with clinically suspected MEN1, we found that MEN1 is most commonly diagnosed in middle-aged adults, with a higher prevalence in females. Despite its rarity, the study provides valuable insights into MEN1 using a large-scale administrative dataset, demonstrating the condition’s significant burden on affected patients. Patients with clinically suspected MEN1 exhibited significant involvement of the parathyroid, pituitary, and duodenopancreatic glands, necessitating multiple surgeries. They had a greater burden of comorbidities. Mortality risk was significantly higher in these patients, particularly in those with multiorgan involvement.

The epidemiology of clinically suspected MEN1 in South Korea shows both similarities and differences with studies conducted in other countries. Current data on MEN1 are very limited, with studies from Japan, the Netherlands, Italy, and France using hospital registries with genetic data but lacking long-term follow-up and clinical outcomes (7–10). The number of patients in these registries ranged from approximately 300 to 1,435 and were collected from large centers across countries. Conversely, our study included 412 patients, indicating that our cohort was not small. However, many individuals in South Korea may still be underdiagnosed. Similar to other countries, the age at diagnosis in Korea is in the 40s, with a higher prevalence among women (1). A key difference was observed in the prevalence of PHPT. Although PHPT is typically the most common manifestation of MEN1, affecting approximately 90% of patients in other cohorts, our study found a lower prevalence (16). This discrepancy likely stems from our stringent operational definition to capture definitive PHPT cases rather than reflecting a true biological difference because of the absence of a specific ICD-10 code for MEN1 (17, 18). Interestingly, pituitary tumors were the second most common manifestation in our cohort contrary to studies from other countries reporting duodenopancreatic neuroendocrine neoplasia (DP-NENs) as the second most frequent manifestation (8, 9, 19). Furthermore, within DP-NENs, gastrinomas and insulinomas were the most frequently identified subtypes in our cohort, whereas non-functioning DP-NENs are generally reported as most common in other studies (20). These discrepancies could be attributed to several factors: potential underdetection of nonfunctioning DP-NENs as well as non-functioning pituitary tumors, limitations in accurately capturing DP-NENs and pituitary tumors through our operational definitions, or a higher prevalence of pituitary tumors in Korean patients with clinically suspected MEN1. Since non-functioning tumors at both sites are often asymptomatic and infrequently require interventions or medications captured by claims data, their true incidence is challenging to estimate reliably within a database-derived cohort. Consequently, careful interpretation of these findings is warranted. These findings highlight the need for further research to elucidate the true prevalence and clinical spectrum of MEN1 expression in the Korean population. Future studies combining nationwide data with genetic testing and detailed clinical information would be invaluable to clarify these epidemiological patterns and potential ethnic variations in MEN1 presentation.

Our study underscores the critical clinical implications of increased comorbidities and mortality in patients with clinically suspected MEN1 compared with those in the general population. Patients with clinically suspected MEN1 exhibit a significantly higher risk for various metabolic syndromes, including diabetes, hypertension, hyperlipidemia, cardiovascular diseases, and malignancies, with a HR for mortality 3.7-fold higher than that of controls. Although no specific studies have investigated the prevalence of comorbidities in patients with clinically suspected MEN1, it has been suggested that such comorbidities might be related to associated endocrine tumors or underlying genetic mutations characteristic of MEN1 (21, 22). Additionally, a higher mortality risk emphasizes the crucial need for early detection and comprehensive management of MEN1-associated conditions (23). Our findings indicate that breast cancer incidence in Korean MEN1 patients is not significantly higher (24). This aligns with international studies, which found no significant increase in breast cancer risk among MEN1 patients in the U.S. and Tasmania, despite a higher relative risk in Dutch patients (24). Factors such as lower body weight and less frequent oral contraceptive use in Korea may contribute to the relatively low breast cancer rates (25, 26). This may explain the small difference between the patient group and the control group in clinically suspected MEN1 in this study. Conversely, we observed notably high incidences of thyroid (40.8%) and pancreatic cancers (32.3%) in our cohort. These findings warrant cautious interpretation. The elevated thyroid cancer prevalence is attributable to detection bias, as MEN1 patients undergo frequent cervical imaging for parathyroid evaluation, leading to incidental identification of thyroid malignancies (27). Similarly, the high rate of pancreatic cancer may reflect misclassification, where aggressive DPNENs characteristic of MEN1 are potentially miscoded as pancreatic adenocarcinomas (20). These observations underscore the need for further research incorporating comprehensive genetic analyses and detailed clinical data to elucidate the true cancer risk profile in MEN1 patients and to understand potential ethnic or regional variations in tumor manifestation (28).

The prevalence of DP-NENs, which is associated with poor prognosis, is particularly significant (29). Although our cohort did not exhibit a high frequency of pancreatic involvement, the mortality associated with these tumors was considerably higher, indicating their severe impact. Management of metastatic DP-NENs in MEN1 patients remains challenging due to their aggressive behavior, complexity of treatment decisions, and limited evidence from prospective trials. Multimodal therapies—including surgical resection, somatostatin analogs, peptide receptor radionuclide therapy, targeted agents such as everolimus or sunitinib, and chemotherapy—are utilized, yet these treatment strategies must be highly individualized, contributing to the difficulty in achieving optimal outcomes and consequently resulting in higher mortality (20). Giannetta et al. (2021) had suggested that systemic inflammatory biomarkers might serve as valuable tools for prognosis and monitoring treatment response in DP-NENs, providing insights beyond conventional clinical and pathological factors (30). Despite advances in diagnosing and treating MEN1-associated tumors, patients with this syndrome continue to have decreased life expectancies compared with the general population, with the mean age of death ranging from 55 to 60 years (31). Our study aligns with these findings, highlighting the severe impact of MEN1 on patient survival and underscoring the need for continuous improvement in early detection and management strategies. Given the complex nature of MEN1 and the range of associated conditions, evidence-based recommendations are still lacking, and many therapeutic options remain controversial, often based on the experiences of certain centers rather than prospective trials (32). This highlights the necessity for a multidisciplinary approach in specialized centers to ensure effective follow-up and management of patients with MEN1. The impact of MEN1 extends beyond physical health and significantly affects patients’ quality of life (HRQOL) and mental health (33, 34). Our findings show that patients with MEN1 experience higher levels of anxiety and depression than controls and face a substantial financial burden that impacts their HRQOL. The Dutch MEN1 study group reported elevated fear of disease occurrence, which correlated with poorer HRQOL scores (35). This emphasizes the need to integrate mental health support into routine clinical management.

This is the first nationwide study of clinically suspected MEN1 in South Korea, and one of the few studies globally to use a nationwide cohort to provide a comprehensive epidemiology of this rare disease. The use of Korean NHIS cohort allowed for long-term follow-up, which was not feasible in many other studies, providing valuable insights into the long-term complications associated with clinically suspected MEN1. This longitudinal approach helps understand the characteristics of clinically suspected MEN1 disease beyond a fragmented view, offering important clues concerning its clinical implications. Moreover, the high mortality rate underscores the significant risks of clinically suspected MEN1, especially in cases requiring hormone management or aggressive interventions. However, the study has several limitations. First, owing to the absence of a specific ICD-10 code for MEN1, we identified patients from the Korean NHIS cohort using an operational definition based on the diagnostic and prescription codes. This method, which is not based on genetic testing, limits the accuracy of determining whether patients truly have MEN1 or ‘genotype-negative MEN1’, which may follow a different clinical course (36, 37). Although our validation in a tertiary center resulted in a high sensitivity (98.9%), the positive predictive value (73.3%) indicates that approximately 1 in 4 patients may be misclassified. Notably, achieving a high PPV in rare disease research is inherently challenging, as demonstrated in previous digital phenotyping studies of other rare endocrine diseases (38). We nevertheless prioritized high sensitivity to capture as many potential MEN1 cases as possible for this rare disease, acknowledging the associated risk of misclassification. Additionally, we did not have access to genetic test results or family history information. The lack of family history data, which is not available in this database, is a significant limitation. Given the limited nationwide research on rare diseases, such attempts remain valuable. Similar methodologies have been employed in previous rare disease studies in Korea, utilizing operational definitions based on diagnostic codes owing to the lack of specific ICD-10 codes (39). Second, selection bias is an inherent limitation. We defined cases based on the presence of hormonal issues or severe symptoms requiring treatment, potentially missing non-functioning cases or those not actively treated by healthcare providers. This bias, caused by the absence of a specific ICD-10 code, could lead to an overestimation of mortality and might impact observations of prevalence in pituitary and duodenopancreatic involvement differently than in other countries. This necessitates careful interpretation of our findings. Additionally, the small number of patients in our study necessitates caution during statistical analysis as this may affect the robustness of our findings. Third, although the classification of single-gland versus multigland parathyroid disease was clinically guided by parathyroidectomy procedure codes (P4541 vs. P4542), variability in clinical coding practices may introduce potential misclassification. This limitation extends to other operational definitions used in this study, as diagnostic and procedural codes inherently carry a risk of inaccurate recording. Lastly, our claims-based definition of adrenal involvement allowed potential duplication across multiple adrenal conditions due to coexisting or co-producing tumors, and the absence of pathological confirmation or hormonal assay data further limits accurate clinical characterization.

In conclusion, this study provides valuable insights into the epidemiology, comorbidity patterns, and clinical outcomes of clinically suspected MEN1 in South Korea. These findings underscore the profound impact of MEN1 on morbidity and mortality and highlight the need for comprehensive management strategies. Despite the inherent limitations of using anonymized nationwide administrative data, such as the inability to include genetic confirmation, this study demonstrates the utility of large-scale population-based cohorts in advancing our understanding of rare diseases like MEN1. Continued research and awareness are essential to optimize the care of patients with MEN1 and improve their quality of life.

## Data Availability

The datasets presented in this study can be found in online repositories. The names of the repository/repositories and accession number(s) can be found below: The data supporting the findings of this study are available from the cohort committees and national registers of the cohorts and countries involved (Korean National Health Insurance Service, https://nhiss.nhis.or.kr).
