# Supplementary material for: Epidemiology and clinical outcomes of clinically suspected multiple endocrine neoplasia type 1 in South Korea: a nationwide cohort study
Source: Front Endocrinol (Lausanne). 2025 Jun 18;16:1562282. doi: 10.3389/fendo.2025.1562282 (PMC12213390; doi:10.3389/fendo.2025.1562282)
Supplement: Supplementary file 1 [file DataSheet1.docx]

**Supplements**

**Epidemiology and Clinical Outcomes of Symptomatic Multiple Endocrine Neoplasia Type 1 in South Korea: a Nationwide Cohort Study**

**Running head:** Epidemiology of MEN1 in South Korea

Kyoung Jin Kim, Min Heui Yu, Yoon-a Hwang, Shinje Moon, Namki Hong, and Yumie Rhee

| **Contents** | **Page** |
| --- | --- |
| **Supplement Table 1.** Definitions and codes used to define key conditions, comorbidities, and drug treatments | 2-4 |
| **Supplement Figure 1.** Flow diagram of participant selection | 5 |
| **Supplement Figure 2.** Age at Diagnosis and Sex Distribution of Patients with Likely Multiple Endocrine Neoplasia Type 1 (MEN1). (A) Age at diagnosis and (B) sex distribution of patients with likely MEN1 | 6 |
| **Supplement Figure 3.** Comparative cumulative incidence of clinical outcomes in patients with MEN1 and control group | 7 |

**Supplement Table 1**. Definitions and codes used to define key conditions, comorbidities, and drug treatments

|  | **Definitions** | **ICD-10 codes or conditions** |
| --- | --- | --- |
| **Clinical manifestations** | | |
| Primary hyperparathyroidism | Defined from at least one diagnostic code with one procedural claim, while meeting the exclusion criteria | ICD-10: E21.0, E21.2, E21.3, E21.4, E21.5, D35.1, D44.2, C75.0  Claim for parathyroidectomy:  (P4541: Parathyroidectomy(Benign)-Single,P4542: Parathyroidectomy(Benign)-Multiple  P4543: Parathyroidectomy(Malignant))  Exclusion Criteria: Any case with at least one CKD diagnostic code (N18-N19, Z94.0) and one procedural code for kidney replacement treatment (Z49.1–49.2, O7072, O7073–7034, O7071, O7072, R3280) within one year before or after a parathyroid diagnosis |
| Pituitary adenoma | Defined from at least one diagnostic code with one procedural claim | ICD-10: C75.1, D35.2, D44.3, E22.0, E22.1, E22.8, E22.9, E24.0  Claims for medications or procedures (bromocriptine, cabergoline, lanreotide, octreotide, pasireotide, surgery for pituitary tumor removal) |
| Duodenopancreatic neuroendocrine tumor | Defined from at least one diagnostic code with one procedural claim  or  At least two instances of the specified diagnostic codes for above accompanied by a liver metastasis diagnosis (C78.7) | ICD-10: E34.0, E34.1, D13.7, C25.4, E16.18, E16.3, E16.4, E16.8, E16.9, C78.7  Claims for medications or procedures (somatostatin analogue or surgery for pancreatectomy) |
| **Comorbidities** | | |
| Diabetes mellitus | Defined from diagnosis plus treatment (≥30 days) at least once | ICD-10: E11–E14  Treatment: various oral antidiabetics (alpha-glucosidase inhibitor, DPP-4 inhibitor, GLP1 agonist, meglitinide, metformin, SGLT2 inhibitor, sulfonylurea, thiazolidinedione) and insulin. |
| Hypertension | Defined from diagnosis plus treatment (≥30 days) at least once | ICD-10: I10–I13, I15  Treatment: various antihypertensive agents (ARB, ACE inhibitors, beta blockers, calcium channel blockers, alpha blockers, diuretics, and others) |
| Dyslipidemia | Defined from diagnosis plus treatment (≥30 days) at least once | ICD-10: 78  Treatment: various lipid lowering agents (statins, fibrates) |
| Osteoporosis | Defined from prescription codes for any treatment received at least once | ICD-10: M80–M82  Treatment: bisphosphonate (alendronate, etidronate, ibandronate, risedronate, pamidronate, zoledronate), denosumab, teriparatide, selective estrogen receptor modulator (bazedoxifene, raloxifene), or hormone replacement therapy (allylestrenol, chlormadinone, dienogest, diethylstilbestrol, dydrogesterone, estradiol, estrogen, ethynylestradiol, levonorgestrel, medroxyprogesterone) |
| Composite cardiocerebrovascular disease | Any event of nonfatal myocardial infarction, cerebrovascular disease, or heart failure | |
| Nonfatal myocardial infarction | Defined from diagnosis with one inpatient or two outpatient records | ICD-10: I21–I23 |
| Nonfatal stroke | Defined from diagnosis with one inpatient or two outpatient records | ICD-10: I60–I69 |
| Heart failure | Defined from diagnosis with one inpatient or two outpatient records | ICD-10: I09.94, I11.0, I13.0, I13.2, I25.5, I42, I43, I50 |
| Fracture complications | Defined from diagnosis with one inpatient or two outpatient records | ICD-10: S52.5, S52.6, S42.2, S42.3, S72.0, S72.1, M48.4, M48.5, M49.5, S22.0, S22.1, S32.0, S82.3, S82.5, S82.6, S82.8, S32.1, S32.2, S32.3, S32.4, S32.5, S32.7, S32.8 |
| Cancer | Defined from diagnosis with one inpatient or two outpatient records | ICD-10: C00–C97  Breast cancer (C50)/ Esophageal cancer (C15)/ Uterus cancer (C54, C55)/ Thymic cancer (C37)/Lung cancer (C34)/ Prostate cancer (C61)/ Colorectal cancer (C18-C20)/Liver cancer (C22)/ Pancreatic cancer (C25)/ Gastric cancer (C16)/ Ovarian cancer (C56)/Cervical Cancer (C53)/ Thyroid cancer (C73)/ Bladder cancer (C67)/ Kidney cancer (C64) |
| **Associated diseases** | | |
| Adrenal involvements | Any event of adrenal adenoma, primary aldosteronism, pheochromocytoma and paraganglioma, or Cushing’s syndrome, or adrenal carcinoma | |
| Adrenal adenoma | Defined from diagnosis with one inpatient or two outpatient records | ICD-10: D35.0, D44.1, E27.9 |
| Primary aldosteronism | Defined from diagnosis with one inpatient or two outpatient records | ICD-10: E260, I1520, I1521 |
| Pheochromocytoma and paraganglioma | Defined from diagnosis with one inpatient or two outpatient records | ICD-10: C74.1 C74.9, I1522, D356, D446, D447, D487, or C755 |
| Cushing's syndrome | Defined from diagnosis with one inpatient or two outpatient records | ICD-10: E24.8, E24.9 |
| Adrenal carcinoma | Defined from diagnosis with one inpatient or two outpatient records | ICD-10: C74.0 |
| Thymus benign tumors | Defined from diagnosis with one inpatient or two outpatient records | ICD-10: D15.0 or D38.4 |
| Lipoma | Defined from diagnosis with one inpatient or two outpatient records | ICD-10: D17.0–9 |
| Meningioma | Defined from diagnosis with one inpatient or two outpatient records | ICD-10: D32.0,1,9 |
| Neoplasm of uncertain or unknown behaviour of brain and central nervous system including ependymoma | Defined from diagnosis with one inpatient or two outpatient records | ICD-10: D43 |
|  |  |  |

ICD-10, International Classification of Diseases, 10th revision.


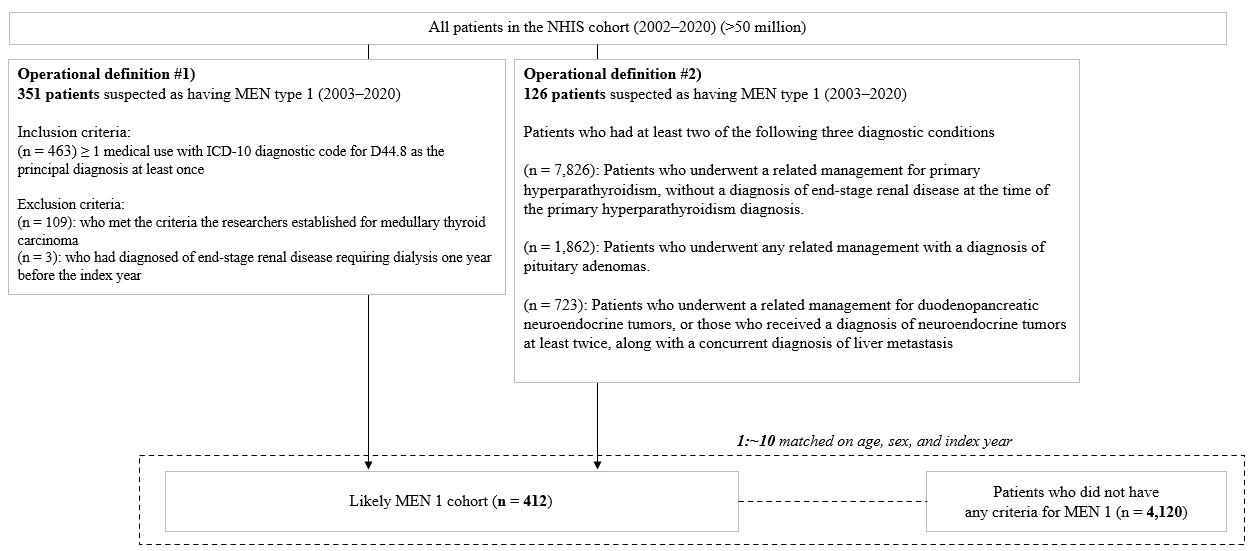
**Supplement Figure 1.** Flow diagram of participant selection


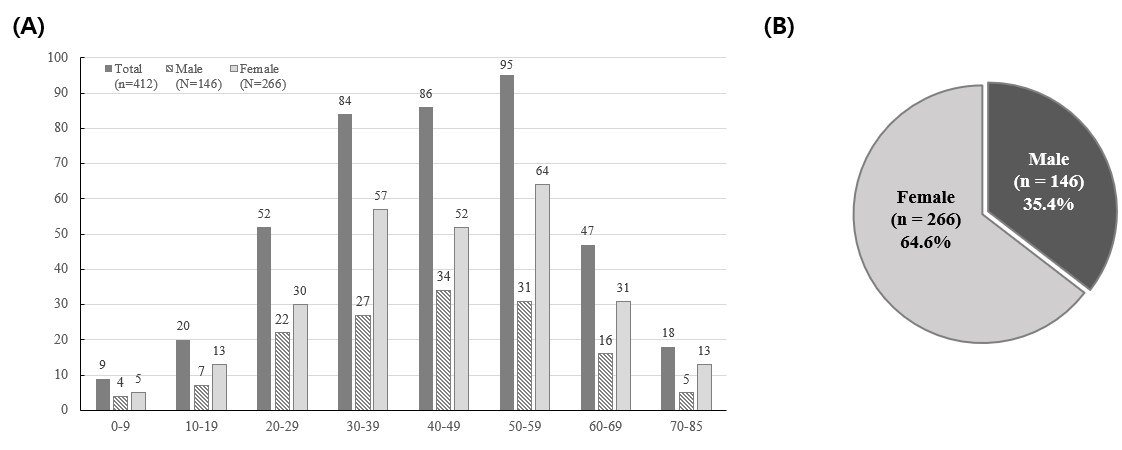
 **Supplement Figure 2**. Age at Diagnosis and Sex Distribution of Patients with Likely Multiple Endocrine Neoplasia Type 1 (MEN1). (A) Age at diagnosis and (B) sex distribution of patients with likely MEN1


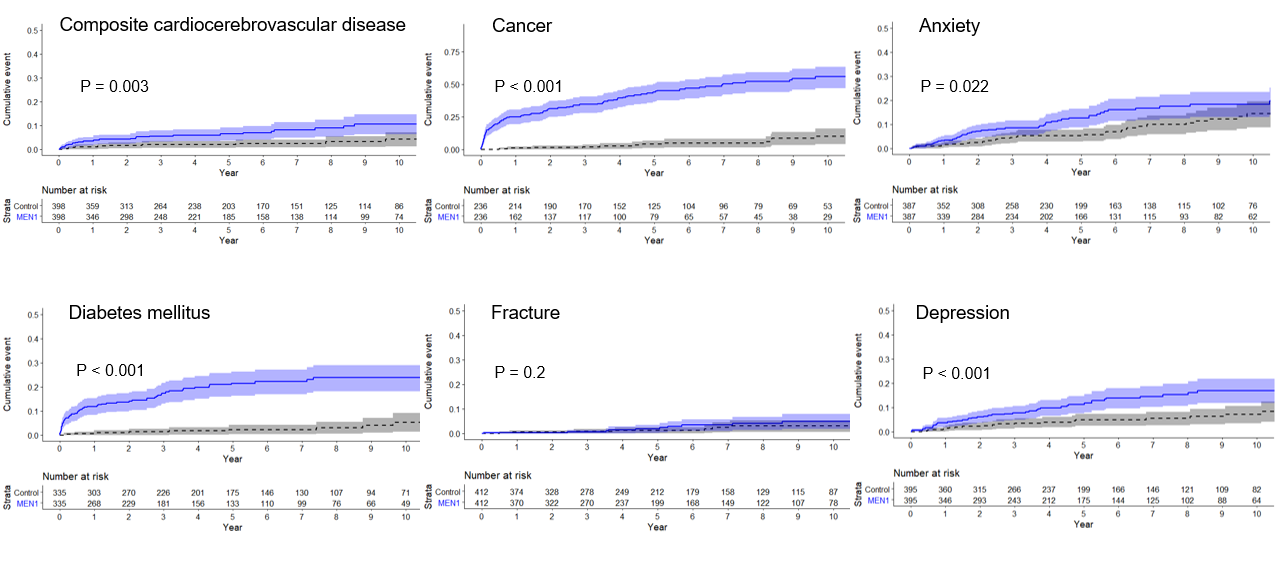
**Supplement Figure 3**. Comparative cumulative incidence of clinical outcomes in patients with MEN1 and control group
